# Water sports could contribute to the translocation of ranaviruses

**DOI:** 10.1038/s41598-019-39674-5

**Published:** 2019-02-20

**Authors:** Rosa Casais, Asier R. Larrinaga, Kevin P. Dalton, Paula Domínguez Lapido, Isabel Márquez, Eloy Bécares, E. Davis Carter, Matthew J. Gray, Debra L. Miller, Ana Balseiro

**Affiliations:** 1 SERIDA, Servicio Regional de Investigación y Desarrollo Agroalimentario, Gijón, Asturias Spain; 2 Enebada Entorno, S.L., Santiago de Compostela, Galicia, Spain; 3 Departamento de Bioquímica, Universidad de Oviedo, Oviedo, Spain; 4 Facultad de Biología, Universidad de León, Campus de Vegazana, León, Spain; 5 Center for Wildlife Health, University of Tennessee Institute of Agriculture, Knoxville, Tennessee United States of America

## Abstract

Ranaviruses have been identified as the cause of explosive disease outbreaks in amphibians worldwide and can be transmitted between hosts both via direct and indirect contact, in which humans might contribute to the translocation of contaminated material. The aim of this study was to evaluate the possible role of water sports in the human translocation of ranavirus, *Batrachochytrium dendrobatidis (Bd)*, and *B*. *salamandrivorans (Bsal)*. A total of 234 boats were sampled during the spring Spanish Canoe Championship which took place in Pontillón de Castro, a reservoir with a history of ranavirosis, in May 2017. Boats were tested for the presence of ranavirus and *Batrachochytrium* spp. DNA, using quantitative real-time polymerase chain reaction techniques (qPCR). A total of 22 swabs (22/234, 9.40%) yielded qPCR-positive results for *Ranavirus* DNA while *Bd* or *Bsal* were not detected in any of the samples. We provide the first evidence that human-related water sports could be a source of ranavirus contamination, providing justification for public disinfecting stations in key areas where human traffic from water sports is high.

## Introduction

Viruses of the genus *Ranavirus* can infect fish, amphibians, and reptiles, impacting biodiversity by causing multi-species declines^1–4^. Ranaviruses have been identified as the cause of explosive disease outbreaks in amphibians worldwide, including in Croatia^5^, Australia^6^, the United Kingdom^7,8^, the United States^9–12^, Canada^13^, Spain^4,14–16^, the Netherlands^17^, France^18^ and Portugal^19^.

Ranaviruses can be transmitted between hosts via direct contact with infected hosts, consumption of infected animals (e.g, predation, necrophagy), or indirectly by contact with virions through exposure to contaminated water, substrates, or fomites^12^. Humans can play a role in the emergence of ranaviral disease (ranavirosis) in animal populations by increasing stressors that subsequently compromise the host immune system, by introducing reservoir species such as fishes and by translocation of a pathogen over large geographic distances often referred to as pathogen pollution^20,21^. Although pathogen pollution of ranavirus by humans through adhesion of virions to boat surfaces or other recreational gear has been suggested^12,21,22^, no studies have demonstrated this so far. Gray *et al*.^23^ recently provided evidence that ranavirus could adhere to examination gloves and hence contribute to transmission among amphibians that are processed as part of a pathogen surveillance study if gloves are not changed between animals. Ranaviruses can co-occur with other pathogens like *Batrachochytrium* spp^19,24–26^, which can also be translocated by human activities. Indeed, humans were to blame for an outbreak of *B*. *dendrobatidis* (*Bd*) when captive animals infected with the pathogen were released as part of a species reintroduction project^27^. In Europe *B*. *salamandrivorans* (*Bsal*) has also been spread through private amphibian trading^28^.

The aim of this study was to evaluate the possible role of water sports in the human translocation of ranavirus and *Batrachochytrium* spp. using molecular techniques. For that purpose boats were sampled during the spring Spanish Canoe Championship which took place in Pontillón de Castro (Spain), a reservoir with a history of ranavirosis.

## Results

### Ranavirus, *Batrachochytrium dendrobatidis* (*Bd*) and *B. salamandrivorans* (*Bsal*) detection

**Figure 1:**
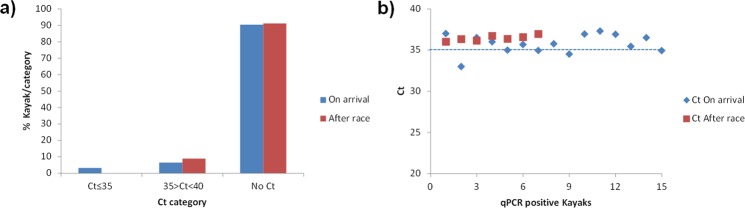
Percentage of kayaks within each ranavirus detection threshold point (Ct) category (**a**), before and after the Spring Spanish Canoe Championship held in the Pontillón de Castro reservoir (Northwestern Spain) in 2017, and Cts obtained in quantitative real-time polymerase chain reaction (qPCR) positive kayaks (**b**). Samples with a cycle threshold (Ct) ≤35 were considered qPCR positives. Samples with a Ct between 35 and 40 were considered weak qPCR positives. Samples with no or no typical amplification curves were regarded as qPCR negative.

**Figure 2:**
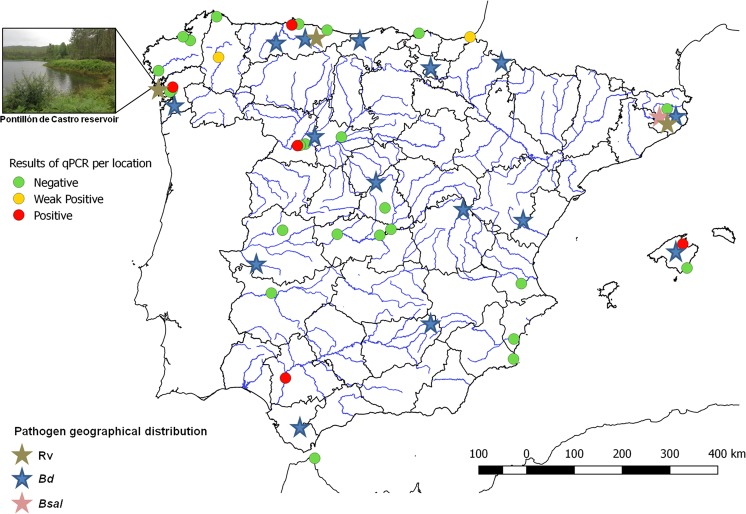
Origin (usual training place) of the participant kayaks in the Spring Spanish Canoe Championship held in the Pontillón de Castro reservoir in 2017, studied for the presence of ranavirus using quantitative real-time polymerase chain reaction (qPCR). Red circles represent training places with at least one positive kayak (threshold point (Ct) ≤35) by qPCR. Yellow circles represent training places with at least one weak positive kayak (Ct between 35 and 40). Green circles represent training places of negative kayaks. Brown, blue and pink stars represent the geographical distribution of Ranavirus (Rv), *Batrachochytrium dendrobatidis* (*Bd*) and *B*. *salamandrivorans* (*Bsal*) in Spain, respectively.

A total of 22 swabs (22/234, 9.40%) yielded quantitative real-time polymerase chain reaction (qPCR)-positive results for ranavirus DNA; five out of the 22 swabs were positives (all at arrival of boats to the racing facilities) and 17 weak positives (10 at water craft arrival and 7 after the race). Percentage (%) of positive and negative kayak per threshold point (Ct) category and the specific Ct of each positive sample are shown in Fig. 1. Regarding the 32 kayaks tested both at arrival and after the race, there were four positive kayaks at arrival that became negative after race while there was one kayak negative at arrival that became positive after race. Positive qPCR DNA samples were also amplified by conventional PCR as described in the Methods section resulting four positive samples. Analysis of the nucleotide sequence of one of the DNA products revealed an identity of 100% with the homologous region of the genome sequence of the common midwife toad ranavirus isolates (GenBank Accession numbers: FM213466, JQ231222 and KJ703124 Spanish isolates from 2007, 2008 and 2011, respectively) and Dutch isolates MF102028-30 (2014–15), MF004271-2 (2011 and 2015), MF062693-5 (2011, 2013 and 2014), MF093732.1 (2014), MF033604.1 (2011), MF038789.1 (2011), KT003477.1 (2010), KP056312.1 (2013). The three water samples from the northern end, middle and southern end of the reservoir yielded positive results for ranavirus DNA (Ct = 33, 34 and 32, respectively). The qPCR-positive kayaks came from different regions of Spain. In Fig. 2 both the origin of the studied kayaks and its result in the qPCR are shown. *Bd* or *Bsal* were not detected in any of the samples.

## Discussion

This research demonstrates for the first time how water-related sports such as kayaking can be a source of pathogen pollution for amphibians. In 2017, more than 50 international and almost 100 Spanish national championships were held in Europe and Spain, respectively^29^. Each championship had an average of 600–800 participants, which provides ample opportunity for pathogen translocation among water bodies by humans if the appropriate control measures (e.g. disinfection) are not taken. Ensuring proper disinfection before and after each championship could aid in stopping the spread of ranavirus, while testing all kayaks on their arrival could be an efficient sampling way to detect new sites where ranavirus presence has remained undetected up to now.

We do not know if the virions on the boat were viable, because qPCR only detects DNA presence, or if there were sufficient quantities of live virus for it to be a source of infection; however, there is the possibility that some virions might remain viable, and present a translocation risk. Further studies will be necessary to confirm the presence of infectious ranavirus particles in the swab samples, i.e. through the isolation of virus in cell culture. Environmental persistence of FV3-like ranaviruses outside the host is probably at least one week^30,31^. Allowing surfaces to dry might facilitate virion inactivation^32^, although some previous studies suggest substantial environmental persistence of ranavirus under dry conditions^33^. The likelihood of contaminated surfaces leading to ranavirus transmission to hosts is likely a result of various environmental and host factors. We would expect that invasion by ranavirus into a population is greatest when highly susceptible species are present, the pathogen is novel to a site (i.e., no coevolution), and host densities are high^34^ – all conditions which may have been fulfilled at Pontillón de Castro. Indeed, ranavirus outbreaks likely occur frequently due to the introduction of the pathogen, starting with only a few infected hosts, and then it becomes amplified as transmission to other individuals occurs rapidly and saturates the population^35^.

Disinfection of boat surfaces and other recreational gear that comes in contact with water represents a reasonable disease management strategy^36^. We found that the number of positive samples was higher at water craft arrival (68.18%) to the reservoir than after the race (22.72%). The sources of the ranaviruses remain unknown as kayaks came from several Spanish regions where ranavirus has not been reported (see Fig. 2). This highlights the need for ranavirus surveillance within these areas. After arrival, approximately 50% of the boats were disinfected with sodium hypochlorite diluted to 5%, which might have contributed to lower detection after the race, in fact four positive kayaks detected as positive at arrival became negative after the race. Future testing should include an additional swab post disinfection, as it is possible that the negative results simply were due to flushing/dilution with water as the boat raced along the waterway. Nevertheless, some post-race detection (seven kayaks were weak positives after race) implies that free-floating ranavirus in Pontillón de Castro, as confirmed by detection of ranavirus DNA in water samples, could have contaminated boats, and emphasizes the need to disinfect watercraft when leaving sites too. Gray *et al*.^36^ provide instructions for application of disinfectants to inactivate ranavirus and chytrid fungi.

In this study, neither *Bd* nor *Bsal* were detected in any of the kayak samples, which suggests that these pathogens have not been introduced into the reservoir or that their survival in or on watercraft is more limited when compared to ranavirus. *Bd* has been found as the cause of amphibian infections in about twenty locations from the north to the south of the Iberian peninsula (http://www.bd-maps.net/), including a protected wetland located 50 kilometers away from Pontillón de Castro^37^ (see Fig. 2). *Bsal* has been detected in the northeast of the Iberian Peninsula^38^. The co-infection of chytridiomycosis and ranavirus has been found in some cases^4,19,25,26^. However, although some studies have observed that infection by one of the pathogens can be followed by co-infection by the other pathogen after a few years^19^, there is not enough evidence to show that there could be a facilitation effect among these pathogens. So far, we have not seen this effect in Pontillón de Castro.

We provide evidence that human-related water sports can be a source of ranavirus contamination. Other activities such as fishing and swimming could be additional sources through contaminated equipment, and need to be investigated. Our results provide justification for public disinfecting stations in key areas where human traffic from water sports is high.

## Methods

### Study area

Pontillón de Castro (23.5 ha, 1.48 hm3) is one of the oldest reservoirs in the region of Galicia (Northwestern Spain). It was built in 1943, enlarged to a depth of 23 meters in 1947, and began providing drinking water to the nearby city of Pontevedra in 1956. In 2007, one end of the reservoir was extended to allow for construction of the Sport Center of Pontillón de Castro, a high level training and competition center for canoeing and kayaking. In that same year, the center currently known as the David Cal Nautical Complex and Race Course, was the venue for the Canoe Sprint European Championships, and has since become a common race course for a wide variety of regional and national championships. Other recreational uses of the reservoir include bathing, fishing and nautical modelling.

The amphibian community of Pontillón de Castro includes at least two urodelan species, the Iberian newt (*Lissotriton boscai*) and marbled newt (*Triturus marmoratus*)^4^, and three anuran species, the Iberian water frog (*Pelophylax perezi*), the Iberian tree frog (*Hyla molleri*) and the common midwife toad (*Alytes obstetricans*; unpublished data). In 2010, shortly after the first confirmed ranavirus-caused amphibian mass mortality in Picos de Europa, Spain^14^, a visitor to the Pontillón de Castro reservoir, located about 500 kilometers from the first Spanish outbreak, reported the presence of dead newts. Since then, recurrent mortality events due to ranavirus have been detected each year, affecting both the Iberian and marbled newts, although never reaching the mortality levels found in 2011, where thousands of newts died^4^.

### Sample collection

We performed our study during the Spring Spanish Canoe Championship (200 and 500 meter races) which took place in the Pontillón de Castro reservoir on May 26th and 27th 2017 and included 500 participants^29^. A total of 234 boats were tested for the presence of ranavirus and *Batrachochytrium* spp. Samples were collected both when boats (either kayaks or canoes) arrived at the David Cal Nautical Complex and Race Course when the boats were still dry (i.e., before entering the complex and prior to a disinfection treatment), and after each race when the boats were still wet. We sampled all participating boats that presented to the disinfection booth upon arrival at the racing facilities (*n* = 154, pre-race sampling). Post-race sampling included boats that passed the weight control at completing of the races (*n* = 80), including 32 that were sampled prior to disinfection and 48 that had not been disinfected prior to the race. Crew members from all boats that were sampled provided names of water bodies on which they had previously raced or trained.

Sterile swabs with plastic shafts were used for sample collection. Sampling was carried out by swabbing the inner and outer part of the hull of boats and all swabs were then placed into individual tubes containing 70% alcohol until processing. Swabbing of the outer face of the hull was done longitudinally, encompassing two passes along both sides, while swabbing of the inner face was focused on corners that retained moisture. Additionally, three water samples (1 liter each and collected into clean containers and stored at 4 °C until processing) were taken from separate locations of the reservoir (at the northern end 100 meters upstream, at the middle and at the southern end 100 meters downstream).

### Detection and identification of ranavirus DNA by Quantitative Real-Time Polymerase Chain Reaction (qPCR)

Swabs from kayaks were processed as described in Gray *et al*.^39^. Briefly, swab samples collected in 70% ethanol were first centrifuged (10 min at 8000 rpm and 4 °C), ethanol was then carefully discarded and the pellet used for genomic DNA extraction using the DNeasy Blood and Tissue kit (Qiagen, Hilden, Germany) following the manufacturer´s instructions but for the fact that DNA samples were obtained in 100 µL of elution buffer. We performed real-time qPCR to determine the presence of ranavirus DNA in the swabs following Picco *et al*.^40^, targeting a 70-bp region of the major capsid protein (MCP), covering nucleotides 97887 to 97956 of the reference genome sequence (Genbank accession number KJ175144). qPCR reactions were set up in a final volume of 20 µl with 5 µM of each primer and 2.5 µM (IDT, Leuven, Belgium), 10 µL Taqman Universal PCR MasterMix (2X) (Thermofisher Scientific, Rockford, IL, USA) and 5 µL of the extracted DNA. The amplification program had a first step of enzyme activation of 10 min at 95 °C, and after that, 40 cycles of amplification were performed with 25 sec at 95 °C and 1 min at 60 °C. Samples were processed in batches of 12, which included reference positive and negative extraction controls in order to discard possible inhibition or contamination events. The positive control consisted of a sample of an adult fresh newt (*Lissotriton boscai*) in which the presence of ranavirus had been confirmed by positive growth in tissue culture, conventional PCR and sequencing, while the negative control consisted of a swab with ethanol. Amplification was carried out in a 7900HT Fast Real-Time PCR System (Applied Biosystems, Thermofisher) and results were analyzed using the Sequence Detection Systems Software version 2.3. Samples with a Ct less than or equal to 35 were considered qPCR positives, while samples giving a Ct of between 35 and 40 were considered weak qPCR positives. Samples with no or no typical amplification curves were regarded as qPCR negative. The qPCR analysis was carried out in two independent runs. A sample was declared positive when it resulted positive or weak positive at least once and the results of the appropriate controls were satisfactory.

Samples positive for viral DNA were also analyzed for the presence of a highly conserved region in the amino terminal of the MCP^41^ using a conventional PCR reaction and the resulting DNA products (530 nucleotides including the primers) were gel purified and sequenced as described in Balseiro *et al*.^14^. The nucleotide sequence was used to carry out a BLAST (basic local alignment search tool) to search for homologous sequences in GenBank.

For qPCR water samples were concentrated by three consecutive centrifugations as follows. They were centrifuged at 8,000 × g for 10 min at 4 °C, the supernatants were discarded and the pellets were homogenized in a total volume of 5 mL of nuclease-free water, which were centrifuged again as described above. The pellets were resuspended in 1 mL nuclease-free water, homogenized for 30 sec in a vortex and centrifuged for 10 min at 16,000 g at 4 °C. Afterwards the pellets were resuspended in 200 µL of nuclease-free water and 50 µL were directly used for DNA extraction and analyzed for the presence of ranavirus DNA by real time qPCR in the same way as for the swabs.

### Detection and identification of *Batrachochytrium dendrobatidis* (*Bd*) and *B*. *salamandrivorans* (*Bsal*) DNA by qPCR

DNA samples were also used in a duplex qPCR for simultaneous detection of *Bd* and *Bsal* as described by Blooi *et al*.^42^ using a StepOne Plus System (Life Technologies). Primers STerF and STerR^43^ and probe STerC were used for *Bsal*. ITS1-3 Chytr, and 5.8 S Chytr primers and TaqMan Chytr MGB2 probe were used for *Bd*^44^. Negative and positive controls were included in each qPCR run. In addition, the presence of inhibition of the amplification step of the qPCR was tested including internal controls. PCR conditions followed Blooi *et al*.^42^ procedure.

## Data Availability

All data is available in the main text.
