# Author Correction: Water sports could contribute to the translocation of ranaviruses

**DOI:** 10.1038/s41598-020-60643-w

**Published:** 2020-02-21

**Authors:** Rosa Casais, Asier R. Larrinaga, Kevin P. Dalton, Paula Domínguez Lapido, Isabel Márquez, Eloy Bécares, E. Davis Carter, Matthew J. Gray, Debra L. Miller, Ana Balseiro

**Affiliations:** 1 SERIDA, Servicio Regional de Investigación y Desarrollo Agroalimentario, Gijón, Asturias Spain; 2 Enebada Entorno, S.L., Santiago de Compostela, Galicia, Spain; 3 Departamento de Bioquímica, Universidad de Oviedo, Oviedo, Spain; 4 Facultad de Biología, Universidad de León, Campus de Vegazana, León, Spain; 5 Center for Wildlife Health, University of Tennessee Institute of Agriculture, Knoxville, Tennessee United States of America

Correction to: *Scientific Reports* 10.1038/s41598-019-39674-5, published online 20 February 2019

The Acknowledgements section in this Article is incomplete.

“This study was partially funded by the Principado de Asturias, PCTI 2018–2020 (GRUPIN: IDI2018-000237) and FEDER. We thank Benjamin Rabanal from the Laboratorio de Técnicas Instrumentales, University of León, for Batrachochytrium spp. PCR analysis, and Frank Pasmans’ Lab for kindly providing Bd and Bsal DNA controls.”

should read:

“This study was partially funded by the Principado de Asturias, PCTI 2018–2020 (GRUPIN: IDI2018-000237) and FEDER. We thank Benjamin Rabanal from the Laboratorio de Técnicas Instrumentales, University of León, for Batrachochytrium spp. PCR analysis, and Frank Pasmans’ Lab for kindly providing Bd and Bsal DNA controls.

Fieldwork was partially funded by the Environment and Land Planning Department of the Regional Government of Galicia (Xunta de Galicia), with the financial support of the European Agricultural Fund for Rural Development (EAFRD) of the European Union. We are indebted to the Galician Canoeing Federation for their help and support during the field work.”

